# Supplementary material for: Clinical Impact of the Use of Warfarin in Patients with Atrial Fibrillation Undergoing Maintenance Hemodialysis
Source: J Clin Med. 2024 Apr 20;13(8):2404. doi: 10.3390/jcm13082404 (PMC11051249; doi:10.3390/jcm13082404)
Supplement: Supplementary file 1 [file jcm-13-02404-s001.zip › Supplementary materials-jcm.pdf]

Supplementary materials

Table S1. Medication types and Health Insurance Review and Assessment Service codes

Table S2. Codes associated with cardiac and cerebrovascular outcomes

Table S3. Other factors associated with all-cause mortality or hemorrhagic stroke

Table S4. Baseline characteristics after propensity score matching

Table S5. Cox regression analyses using cohort after propensity score matching

Figure S1. Study flowchart

Figure S2. Distribution of propensity score before and after matching

Table S1. Medication types and Health Insurance Review and Assessment Service codes

**Medications**	**Codes**
Atorvastatin	111502ATB, 502202ATB, 633900ATB, 472400ATB, 518900ATB, 524100ATB, 527000ATB, 672000ATR, 672100ATR, 111503ATB, 502203ATB, 634800ATB, 472500ATB, 111504ATB, 502204ATB
Fluvastatin	162401ACH, 162402ACH, 162403ATR
Lovastatin	185801ATB
Pitavastatin	470901ATB, 470902ATB, 470903ATB
Pravastatin	216601ATB, 216602ATB, 216603ATB, 216604ATB
Rosuvastatin	454001ATB, 454002ATD, 454002ATB, 454003ATB, 454003ATD, 454005ATB
Simvastatin	227801ATB, 227802ATB, 227803ATB, 227805ATB, 227806ATB
Valsartan + Pitavastatin	634900ATB, 635000ATB, 635100ATB, 635200ATB
Valsartan + Rosuvastatin	629700ATB, 629800ATB, 525000ATB, 525100ATB, 525200ATB, 525300ATB,
Olmesartan + Rosuvastatin	653200ATB, 644100ATB, 644200ATB, 526300ATB, 526400ATB, 526500ATB, 526900ATB
Telmisartan + Rosuvastatin	629900ATB, 630000ATB, 630100ATB, 630200ATB, 631600ATB, 631700ATB
Telmisartan + Rosuvastatin + Amlodipine	671700ATB, 671600ATB, 671500ATB, 671400ATB, 671300ATB, 671200ATB,
Losartan + Rosuvastatin + Amlodipine	663900ATB, 664000ATB, 664100ATB, 664200ATB, 664300ATB, 664400ATB,
Fimasartan + Rosuvastatin	654600ATB, 654700ATB, 654800ATB, 654900ATB, 655000ATB
Candesartan + Rosuvastatin	673700ATB, 661800ATB, 661900ATB, 662000ATB, 662100ATB
Irbesartan + Atorvastatin	527000ATB, 527100ATB, 524000ATB, 524100ATB
Atorvastatin + Ezetimibe	633800ATB, 633900ATB, 634800ATB
Pitavastatin + Fenofibrate	679300ACH
Rosuvastatin + Ezetimibe	640700ATB, 640800ATB, 640900ATB
Amosulalol	107901ATB, 107902ATB
Arotinolol	110202ATB, 110201ATB
Atenolol	483102ATB, 111402ATB, 483101ATB, 111403ATB, 111401ATB
Atenolol + Chlorthalidone	262100ATB, 460200ATB
Betaxolol	116801ATB, 116803ATB
Bevantolol	117002ATB, 117001ATB
Bisoprolol	117904ATB, 117903ATB, 117902ATB, 117901ATB
Bisoprolol + Hydrochlorothiazide	469800ATB, 470000ATB, 469900ATB
Carteolol	124801ATB
Carvedilol	125005ATB, 125003ATB, 662201ATB, 125008ACR,

	125001ATB, 662202ATB, 125007ACR, 125002ATB, 125006ACR, 125004ACR
Celiprolol	129101ATB
Metoprolol	194003ATR, 193802ATB, 262400ATR
Metoprolol + Hydrochlorothiazide	262600ATB
Metoprolol + Felodipine	262400ATR
Nadolol	198301ATB
Nebivolol	489501ATB, 489502ATB, 489503ATB
Propranolol	219901ATB, 219904ATB, 219906ACR, 219905ACR
Aspirin	110701ATB, 110702ATB, 110801ATB, 110802ATB, 111001ACE, 111001ATB, 111001ATE, 111002ATE, 111003ACE, 111003ATE
Clopidogrel	133201ACR, 133201ATB, 133201ATR, 133202ATB, 133203ATR, 506100ATB
Aspirin + Bethocarbamol	256800ATB
Aspirin + Clopidogrel	517900ACH, 517900ACE, 517900ATE, 667500ACE
Aspirin + Dipyridamole	489700ACR
Warfarin	249103ATB, 249105ATB
Dabigatran	613701ACH, 613702ACH
Rivaroxaban	511401ATB, 511402ATB, 511403ATB, 511404ATB
Apixaban	617001ATB, 617002ATB
Edoxaban	643601ATB, 643602ATB, 643603ATB

Table S2. Codes associated with cardiovascular events.

**ICD-10 codes**	
Myocardial infarction	I21–I23
Stroke	I60–I63
**Procedure or operation codes**	
Percutaneous coronary intervention	M6551, M6552, M6561–M6564, M6571, M6572, M6601, M6602
Coronary artery bypass grafting	O1641, O1642, O1647, OA641, OA642, OA647
**Medical treatment codes**	
Protein C	635801BIJ
Tissue-type plasminogen activator	223501BIJ, 223502BIJ
Tenecteplase	450302BIJ, 450301BIJ
Tirofiban	240201BIJ, 240230BIJ
Urokinase	246401BIJ, 246405BIJ, 246407BIJ, 246404BIJ, 246406BIJ

Table S3. Other factors associated with all-cause mortality or hemorrhagic stroke

	All-cause mortality				Hemorrhagic stroke			
	Univariate		Multivariable		Univariate		Multivariable	
	**HR (95% CI)**	***P***	**HR (95% CI)**	***P***	**HR (95% CI)**	***P*-value**	**HR (95% CI)**	***P*-value**
Age (increase in 1 year)	1.06 (1.05–1.06)	<0.001	1.05 (1.05–1.06)	<0.001	1.01 (0.99–1.02)	0.387	1.00 (0.99–1.01)	0.934
Sex (ref: male)	0.97 (0.90–1.05)	0.438	0.85 (0.78–0.93)	<0.001	1.11 (0.87–1.43)	0.397	0.97 (0.72–1.31)	0.845
BMI (increase in 1 kg/m^2)	0.97 (0.96–0.98)	<0.001	0.96 (0.95–0.97)	<0.001	0.94 (0.90–0.97)	<0.001	0.93 (0.89–0.97)	<0.001
Vascular access type (ref: AVF)	1.27 (1.15–1.39)	<0.001	1.05 (0.96–1.16)	0.276	1.20 (0.88–1.62)	0.247	1.12 (0.82–1.52)	0.489
Diabetes	1.38 (1.28–1.49)	<0.001	1.35 (1.24–1.46)	<0.001	1.38 (1.08–1.77)	0.010	1.51 (1.14–1.99)	0.004
HD vintage (increase in 1 M)	0.99 (0.99–1.00)	0.810	1.01 (1.00–1.01)	<0.001	1.00 (0.99–1.00)	0.747	1.00 (0.99–1.00)	0.816
CCI score (increase in 1 unit)	1.08 (1.07–1.10)	<0.001	1.03 (1.02–1.05)	<0.001	1.05 (1.01–1.09)	0.042	1.02 (0.98–1.07)	0.342
UFV (increase in 1 kg/session)	0.90 (0.87–0.94)	<0.001	1.08 (1.03–1.13)	0.002	0.93 (0.81–1.06)	0.273	0.95 (0.82–1.11)	0.518
Kt/V_{urea} (increase in in 1 unit)	0.92 (0.79–1.08)	0.336	0.69 (0.57–0.84)	<0.001	1.41 (0.87–2.27)	0.165	1.15 (0.63–2.08)	0.651
Hb (increase in 1 g/dL)	0.90 (0.86–0.94)	<0.001	0.93 (0.89–0.97)	0.002	0.85 (0.74–0.99)	0.033	0.86 (0.74–1.00)	0.054
Albumin (increase in 1 g/dL)	0.44 (0.39–0.50)	<0.001	0.64 (0.56–0.73)	<0.001	0.84 (0.58–1.22)	0.354	0.86 (0.58–1.29)	0.472
Cr (increase in 1 mg/dL)	0.89 (0.88–0.90)	<0.001	0.95 (0.94–0.97)	<0.001	0.96 (0.92–1.01)	0.080	0.98 (0.92–1.05)	0.585
Phos (increase in 1 mg/dL)	0.90 (0.87–0.92)	<0.001	1.07 (1.04–1.11)	<0.001	1.01 (0.92–1.10)	0.882	1.08 (0.98–1.20)	0.134
Calcium (increase in 1 mg/dL)	0.97 (0.92–1.02)	0.202	1.08 (1.03–1.14)	0.003	1.17 (1.00–1.36)	0.047	1.20 (1.02–1.41)	0.024
Clopidogrel (ref: non-user)	1.36 (1.25–1.47)	<0.001	1.19 (1.09–1.29)	<0.001	1.13 (0.87–1.48)	0.359	1.14 (0.86–1.52)	0.349
Aspirin (ref: non-user)	1.12 (1.04–1.21)	0.004	1.03 (0.95–1.12)	0.440	1.05 (0.82–1.34)	0.728	1.08 (0.83–1.40)	0.558
Satins (ref: non-user)	1.08 (0.99–1.16)	0.061	0.95 (0.87–1.03)	0.250	1.06 (0.83–1.36)	0.635	1.04 (0.79–1.36)	0.781
β-blockers (ref: non-user)	0.98 (0.91–1.06)	0.686	1.01 (0.94–1.09)	0.761	1.15 (0.90–1.46)	0.281	1.08 (0.84–1.38)	0.571
$CHA_2DS_2\text{-}VASc_C$ score	1.10 (1.08–1.13)	<0.001	–	–	1.12 (1.04–1.21)	0.004	–	–

HAS-BLED score	1.35 (1.30–1.40)	<0.001	–	–	1.16 (1.04–1.31)	0.010	–	–

Multivariate analysis was adjusted for the use of warfarin; age; sex; BMI; vascular access type; HD vintage; diabetes; CCI score; UFV; Kt/V_{urea}; Hb, serum albumin, serum Cr, Phos, and serum calcium levels; and the use of statins, clopidogrel, aspirin, or β-blockers and was performed using enter mode.

Abbreviations: AV, arteriovenous fistula; BMI, body mass index; CCI, Charlson Comorbidity Index; CI, confidence interval; Cr, creatinine; Hb, hemoglobin; HD, hemodialysis; HR, hazard ratio; Phos, phosphorus; UFV, ultrafiltration volume

Table S4. Baseline characteristics after propensity score matching

	No group (n = 4,009)	Warfarin group (n = 820)	*P*-value
Age (years)	67.0 ± 11.4	66.9 ± 10.9	0.888
Sex (male, %)	1015 (62.6%)	527 (64.3%)	0.450
Hemodialysis vintage (days)	52 ± 58	53 ± 59	0.812
Body mass index (kg/m^2)	22.4 ± 3.6	22.4 ± 3.4	0.934
Underlying cause of ESKD (diabetes)	919 (56.7%)	363 (44.3%)	0.682
CCI score	8.8 ± 2.9	8.8 ± 2.7	0.942
Kt/V_{urea}	1.52 ± 0.24	1.51 ± 0.25	0.732
UFV (L/session)	2.3 ± 0.9	2.3 ± 0.8	0.890
Hemoglobin (g/dL)	10.7 ± 0.9	10.7 ± 0.9	0.536
Serum albumin (g/dL)	3.92 ± 0.35	3.91 ± 0.33	0.504
Serum phosphorus (mg/dL)	4.7 ± 1.3	4.7 ± 1.3	0.946
Serum calcium (mg/dL)	8.9 ± 0.8	8.9 ± 0.8	0.400
Serum creatinine (mg/dL)	8.6 ± 2.5	8.7 ± 2.5	0.887
Use of aspirin	627 (38.7%)	319 (38.9%)	0.950
Use of clopidogrel	298 (18.4%)	138 (16.8%)	0.373
Use of statins	602 (37.1%)	396 (48.3%)	<0.001
Use of β-blockers	858 (52.9%)	450 (54.9%)	0.385

Data are expressed as mean ± standard deviation for continuous variables and as numbers (percentages) for categorical variables. *P*-values are determined using a t-test, and Pearson's χ^2 test was performed for categorical variables. Abbreviations: CCI, Charlson Comorbidity index; ESKD, end-stage kidney disease; UFV, ultrafiltration volume

Table S5. Cox regression analyses using cohort after propensity score matching

	Univariate		Multivariable	
	**HR (95% CI)**	***P***	**HR (95% CI)**	***P***
All-cause mortality	1.15 (1.03–1.29)	0.016	1.14 (1.02–1.28)	0.025
Ischemic stroke	0.96 (0.74–1.25)	0.777	0.96 (0.74–1.24)	0.749
Hemorrhagic stroke	1.74 (1.23–2.47)	0.002	1.76 (1.24–2.50)	0.001
Cardiovascular events	0.94 (0.73–1.22)	0.643	0.95 (0.73–1.23)	0.678

Adjustments in multivariable analysis included body mass index, vascular access type, age, sex, diabetes, Charlson Comorbidity Index score, hemodialysis vintage, ultrafiltration volume, Kt/V_{urea}, serum albumin, hemoglobin, serum calcium, serum creatinine, serum phosphorus, systolic blood pressure, diastolic blood pressure, and use of anti–hypertensive drug, aspirin, and statins. The analysis was conducted using the enter mode. Abbreviations: CI, confidence interval; HR, hazard ratio.

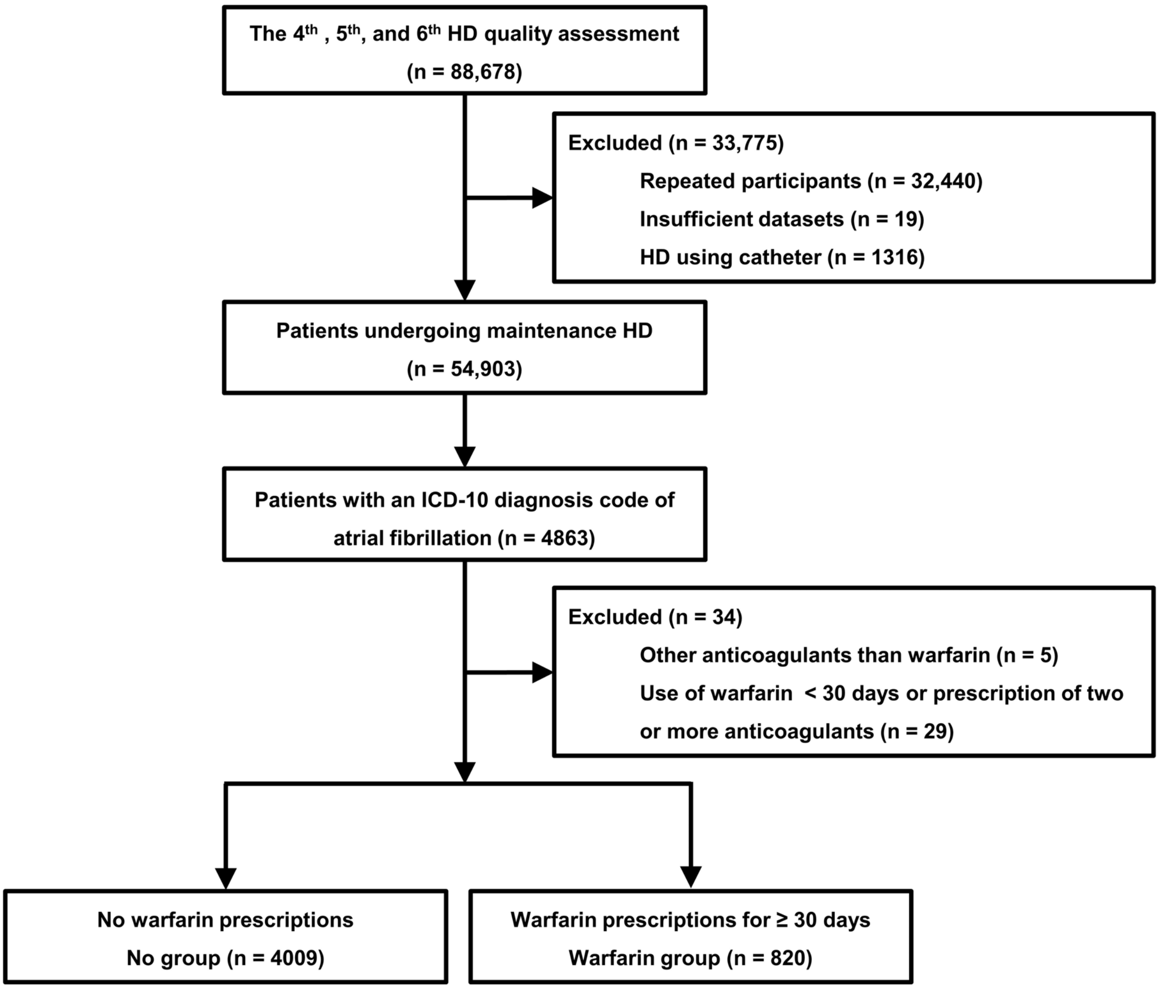

Figure S1. Study flow chart.

Abbreviations: HD, hemodialysis; ICD-10, International Classification of Disease-10th version.

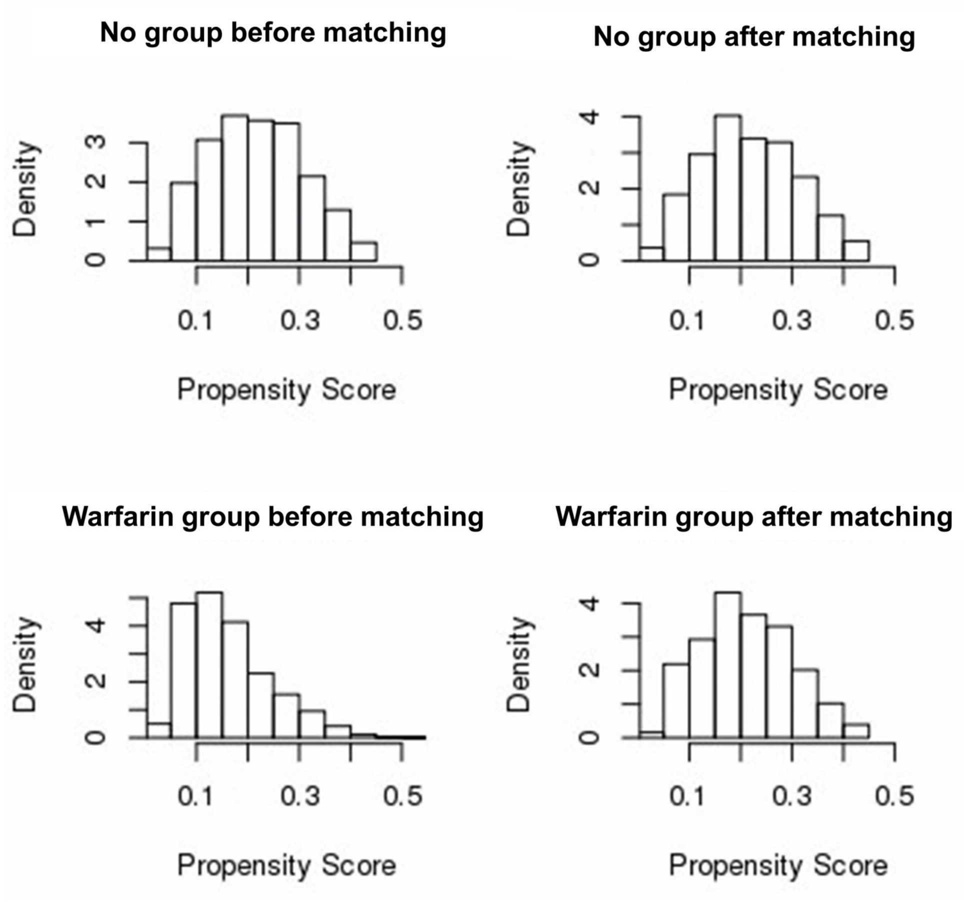

Figure S2. Distribution of propensity score before and after matching.
